# *Vitex agnus-castus* L. (Verbenaceae) Improves the Liver Lipid Metabolism and Redox State of Ovariectomized Rats

**DOI:** 10.1155/2015/212378

**Published:** 2015-04-14

**Authors:** Franciele Neves Moreno, Lilian Brites Campos-Shimada, Silvio Claudio da Costa, Rosângela Fernandes Garcia, Alessandra Lourenço Cecchini, Maria Raquel Marçal Natali, Adriana de Souza Vitoriano, Emy Luiza Ishii-Iwamoto, Clairce Luzia Salgueiro-Pagadigorria

**Affiliations:** ^1^Laboratory of Biological Oxidations, Department of Biochemistry, University of Maringá, 87020900 Maringá, PR, Brazil; ^2^Laboratory of Center of Natural Products, Department of Biochemistry, University of Maringá, 87020900 Maringá, PR, Brazil; ^3^Department of Physiological Sciences, University of Maringá, 87020900 Maringá, PR, Brazil; ^4^Department of General Pathology, University of Londrina, 86057970 Londrina, PR, Brazil; ^5^Department of Morphological Sciences, University of Maringá, 87020900 Maringá, PR, Brazil

## Abstract

*Vitex agnus-castus* (VAC) is a plant that has recently been used to treat the symptoms of menopause, by its actions on the central nervous system. However, little is known about its actions on disturbances in lipid metabolism and nonalcoholic fat liver disease (NAFLD), frequently associated with menopause. Ovariectomized (OVX) rats exhibit increased adiposity and NAFLD 13 weeks after ovary removal and were used as animal models of estrogen deficiency. The rats were treated with crude extract (CE) and a butanolic fraction of VAC (ButF) and displayed the beneficial effects of a reduction in the adiposity index and a complete reversion of NAFLD. NAFLD reversion was accompanied by a general improvement in the liver redox status. The activities of some antioxidant enzymes were restored and the mitochondrial hydrogen peroxide production was significantly reduced in animals treated with CE and the ButF. It can be concluded that the CE and ButF from *Vitex agnus-castus* were effective in preventing NAFLD and oxidative stress, which are frequent causes of abnormal liver functions in the postmenopausal period.

## 1. Introduction

The postmenopausal state is a period in woman's life that is accompanied by profound disturbances in lipid metabolism and a higher incidence of metabolic syndrome (MS) [1]. In the liver, estrogen deficiency results in the accumulation of triacylglycerols (TG) in the cytosol of hepatocytes, a characteristic of nonalcoholic fatty liver disease (NAFLD) [2–4]. It is believed that estrogen directly and positively influences the hepatic lipid metabolism via genomic actions [5]. The fatty acid accumulation in the liver is also known to be associated with oxidative cell damage [6].

Hormone replacement therapy with estrogen or estrogenic compounds has proven to be effective in treating various MS disorders, including NAFLD, both in postmenopausal women [2] and in ovariectomized (OVX) rodents [3, 4]. The recognized benefits and associated risks of conventional hormone therapy have intensified the search for new compounds with estrogenic properties, such as synthetic steroids and phytoestrogens. Thus, pre- and postmenopausal women are becoming adept at using alternative hormonal therapies, such as herbal compounds or medicinal plants [7].

*Vitex agnus-castus* L. (Verbenaceae) (VAC) [8] has long been used as an herbal remedy for menstrual cycle irregularities and premenstrual syndrome and more recently for the treatment of menopause symptoms, such as hot flushes and sleep disturbances [9, 10]. Most of the VAC used by women comes from food supplements obtained from hydroalcoholic plant extracts, which have high amounts of the two glycosylated iridoids aucubin and agnuside [11]. The extraction of crude extract with* n*-butanol is able to concentrate agnuside (AGN) [11], to which has been attributed many of the pharmacological properties of VAC, including dopaminergic activity [9].

Despite the beneficial effects of VAC on the central nervous system [9, 10], little is known about its effects on lipid metabolism and the redox state of the liver, particularly in the postmenopausal condition.

Much of our current view of the actions of estrogenic compounds on adiposity and the metabolic disturbances associated with menopause is derived from the studies with OVX rodents. Thus, the aim of the present work was to evaluate whether treating OVX rats with a crude hydroalcoholic extract of VAC (CE) or a butanolic fraction of VAC (ButF) protects the liver against the lipid-based metabolic abnormalities and cellular oxidative stress commonly associated with postmenopausal conditions [1, 3–5, 12–14]. Several parameters were measured, including the content of liver lipids, mitochondrial and peroxisomal fatty acid oxidation, mitochondrial reactive oxygen species (ROS) generation, and the activities of the most important enzymatic and nonenzymatic ROS scavenger systems. Body weight gain, adiposity, and biochemical parameters of plasma were also evaluated.

## 2. Experimental Procedures 

### 2.1. Material

The following substrates and reagents were purchased from Sigma Chemical Co. (St. Louis, USA): 2,4-dinitrophenol (DNP), phenylmethylsulfonylfluoride (PMSF), reduced glutathione (GSH), oxidized glutathione (GSSG), sodium dodecyl sulfate (SDS), aminotriazole (3-amino-1,2,4-triazole), horseradish peroxidase, 2′,7′-dichlorofluorescein diacetate (DCFH-DA), 2′,7′-dichlorofluorescein (DCF),* o*-phthalaldehyde (OPT), Brij L23 solution, 1-palmitoyl-*sn*-glycero-3-phosphocholine, oxidized 3-acetylpyridine adenine dinucleotide (APAD), Sephadex LH-20, and 2,4-dinitrophenylhydrazine (DNPH). Kits from Gold Analisa (Belo Horizonte, Brazil) were used to measure plasma lipid and glucose levels. Sodium heparin was obtained from Roche. The other reagents used were from Merck (Darmstadt, FRG), Carlo Erba (São Paulo, Brazil), and Reagen (Rio de Janeiro, Brazil).

### 2.2. Crude VAC Extract (CE)

The hydroalcoholic, dried fruit extract of* Vitex agnus-castus* L. was acquired from GAMMA Trade, Import & Export Ltd. (Lot #110610, São Paulo, SP, Brazil) and stored in the dark in a cool, dry place until use. From this crude extract (CE) was obtained the butanolic fraction of VAC (ButF).

### 2.3. Preparation of Butanolic Fraction of VAC (ButF)

A portion of the CE (9.9 g) was subjected to column chromatography using Sephadex LH-20 (50 g; 2.5 × 30 cm) and methanol. One hundred fractions were obtained (15 mL each, flow rate of 3.0 mL/min). Fractions 2–45 were combined and dried via evaporation. Then, the residue (1.891 g) was redissolved in 30 mL distilled H_2_O and partitioned in 60 mL of each of the following solvents: hexane, ethyl ether, and* n*-butanol. The solvents were evaporated and produced: 0.008 g in the hexane fraction, 0.026 g in the ethyl ether fraction, and 0.2577 g in the butanolic fraction. The butanolic fraction was used in the assays.

### 2.4. HPLC Analysis

Chromatographic analysis was performed using a Gilson model 307 liquid chromatography system with a Rheodyne-type injection valve, a 20 *μ*L sample capacity, and UV/VIS detector brand Gilson model 151. Separation was performed on a Thermo BDS Hypersil C18 column (250 × 4.6 mm; 5 *μ*m particle size) with a mobile phase of methanol: H_2_O (80 : 20, v/v) in an isocratic mode, at a flow rate of 0.5 mL/min. The sample injection volume was 10 *μ*L, and the column temperature was controlled at room temperature (22 ± 3°C). AGN (Sigma, USA; Lot #BCBL9655V, purity ≥ 95%) at concentrations of 0.5 and 1.0 mg/mL was used as external standard. A positive identification of AGN in the samples was accomplished by comparing standard retention time at 254 nm. The CE and the ButF were dissolved in 10 mL of 80% methanol (1 mg/mL) and filtered using qualitative filter papers (50 × 50 cm 80 g).

The percentage of AGN in the extracts was calculated from the standard AGN area as below:

(1) Sample  area(1.0 mg/mL)AGN  standard  area(1.0 mg/mL)×100% =amount  of  AGN%.

**Figure 1:**
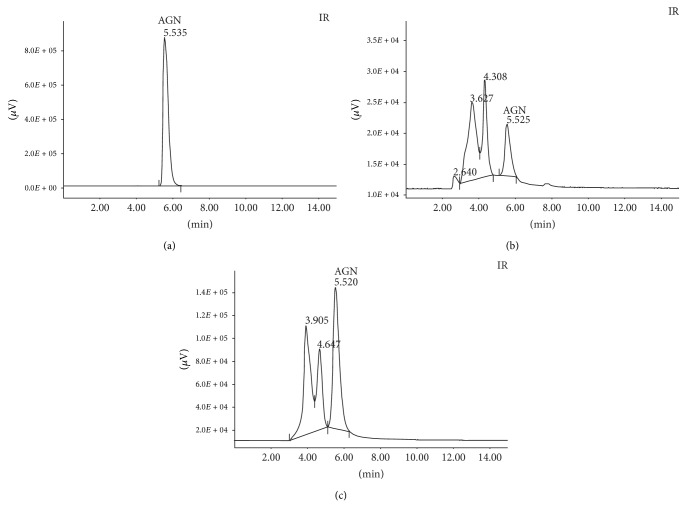
High performance liquid chromatography (HPLC) of standard AGN (Panel (a)) CE (panel (b)) and ButF (panel (c)). HPLC traces of 10 *μ*g standard AGN, CE, or ButF. The retention times of AGN and other majors peaks measured at 254 nm are indicated.

In Figure 1 the chromatographic profiles of the standard AGN are presented (panel (a)), CE (panel (b)) and ButF (panel (c)). The retention time of standard AGN was 5.535 min. The peak of AGN in CE and ButF was, respectively, at 5.525 min and 5.520 min. From these profiles, it was found that commercial extract (CE) presented 0.54% of AGN and the butanolic fraction (ButF) presented 8.75% of AGN.

### 2.5. Animals

Groups of female Wistar rats (45 days old), weighing from 130 to 160 g were provided by the central biotery of the University of Maringá and were randomly assigned to one of two surgical procedures:* sham*-operated (control) and bilateral ovariectomy (OVX). Food consumption and animal weights were measured throughout the entire experimental period (13 weeks). During this period, the rats were maintained in polypropylene cages (maximum of four animals per cage), fed with standard diet and water* ad libitum* and kept in a sectorial biotery at controlled temperature (23°C) and a light/dark cycle of 12 h. All experiments were conducted in strict adherence to the guidelines of the Ethics Committee for Animal Experimentation of the University of Maringá (certificate number 050/2011).

### 2.6. Surgical Procedures

For the surgical procedure, the female rats (45 days old) were divided into two groups: OVX and control animals (in a proportion of 3 : 1). The rats were anesthetized (10 mg xylazine + ketamine 50 mg/Kg,* i.p.*) and had their ovaries removed. The control animals were submitted to the same procedures without removing the ovaries.

### 2.7. Animal Treatment and Material Collection

Ten weeks after the surgical procedures, the animals were treated via oral gavage with daily doses of the CE or ButF suspended in Arabic gum (1%) over a period of 3 weeks. The OVX group was randomly subdivided into three groups: untreated rats (OVX rats that received vehicle), rats treated with daily doses of the CE (8.33 mg/Kg body wt - OVX + CE), and rats treated with daily doses of the ButF (0.83 mg/Kg body wt- OVX + ButF). The animals were treated with CE dosages similar to those recommended for postmenopausal women and the ones treated with ButF received AGN in amounts close to the maximum recommended (0.073 mg/Kg AGN) [15]. On the day of the experiments, the animals were anesthetized with thiopental sodium (50 mg/kg* i.p.*) to collect blood samples and to remove the liver, adipose deposits, and uterus.

### 2.8. Adiposity Index

The retroperitoneal, uterine, mesenteric, and inguinal fat deposits were weighed and expressed in g per 100 g of body weight (BW). The adiposity index was calculated from the sum of the weights of these tissues and was expressed in g per 100 g BW. The uterus was also collected, weighed, and expressed in g per 100 g of BW to confirm the success of OVX.

### 2.9. Serum and Plasma Biochemical Analysis

Blood was collected by cardiac puncture to obtain serum and plasma from fasted rats. Total cholesterol, high-density lipoprotein (HDL-cholesterol), TG, and free fatty acids (FFA) were analyzed in serum and glucose in plasma by standard methods using assay kits (Gold Analisa). Very low density lipoprotein (VLDL-cholesterol) levels were calculated using the Friedewald equation, and low-density lipoprotein (LDL-cholesterol) levels were determined by subtracting HDL and VLDL cholesterol from total cholesterol.

### 2.10. Liver Histochemical Analysis and Lipid Content Determination

Liver fragments were processed by sampling from each batch of animals and stained with Sudan III, which specifically detects lipids, according to the technique described by Pearse [16] to prove the existence of steatosis. The liver total lipid content was determined using the gravimetric method described by Folch et al. [17], which is based on the extraction of lipids from homogenized liver fragments (approximately 0.5 g) in a chloroform-methanol mixture (2 : 1) and expressed as percentage (g/100 g wet liver weight).

### 2.11. Isolation of Liver Fractions

Liver mitochondria were isolated by differential centrifugation in a mannitol-sucrose medium, as described by Bracht et al. [18]. The livers were chopped and homogenized using a Dounce homogenizer, in medium containing 200 mM mannitol, 76 mM sucrose, 0.2 mM EGTA, 0.1 mM PMSF, 1.0 mM Tris (pH 7.4), and 50 mg/100 mL fatty acid-free bovine serum albumin (w/v). The homogenate was fractionated via sequential centrifugation at 536 ×g and 7,080 ×g for 10 min each. The supernatants were used to isolate peroxisomes (postmitochondrial supernatants) and the sediments containing the mitochondria were washed twice by suspension and centrifugation at 6,392 ×g. The final mitochondrial pellet was suspended in a small volume of isolation medium to yield a protein concentration of 40–50 mg/mL.

Intact mitochondria were used to measure *β*-oxidation capacity, ROS generation, and nicotinamide nucleotide transhydrogenase (NNT) activity. Freeze-thaw disrupted mitochondria were used for measurements of GSH contents. To measure the activities of the antioxidant enzymes glutathione peroxidase 1 (GPx1), glutathione reductase (GR), and manganese-superoxide dismutase (MnSOD), mitochondria were disrupted by sonication according to the following procedure: the fraction containing approximately 50 mg of protein/mL was diluted 1 : 10 (v/v) with 0.1 M KCI and 20 mM Tris-HCI, pH 7.4, and treated for 60 seconds in a sonicator tuned to maximum power output. Aliquots of these fractions (1.0 mL) were transferred to microcentrifuge vials and centrifuged at 6,000 ×g for 10 min to sediment intact mitochondria [19]. The activities of the enzymes listed above were measured in the supernatant using specific, classical methods as described below.

Liver peroxisomes were isolated by centrifugation of the postmitochondrial supernatants [20] at 15,000 ×g for 5 min and then at 39,000 ×g for 10 min. These sediments were resuspended and homogenized with a Dounce homogenizer in cold medium containing 250 mM sucrose, 1 mM EDTA, 10 mM Tris (pH 7.3), and 0.1 mM PMSF and were centrifuged again at 15,000 ×g for 10 min to remove mitochondrial contamination. Next, the supernatant was centrifuged at 39,000 ×g for 10 min to obtain the purified peroxisomal fraction, which was resuspended and homogenized to yield a final protein concentration of approximately 20 mg/mL. Peroxisomes were used to measure the *β*-oxidation capacity and catalase (CAT) activity.

Homogenates obtained from freeze-clamped liver of overnight fasted rats were used for GSH content and thiobarbituric acid reactive substances (TBARS) measurements. Pieces of approximately 1.0 g were clamped in liquid nitrogen and homogenized with a Van Potter device in a medium containing 250 mM sucrose, 1 mM EGTA, and 10 mM HEPES, pH 7.2. These homogenates (approximately 50 mg/mL) were used for TBARS measurements. For GSH measurements, this homogenate was centrifuged at 10,000 ×g for 3 min after diluting 25 *μ*L in 1.5 mL of a precipitating medium containing 125 mM sucrose, 65 mM KCl, 10 mM HEPES, pH 7.4, and 15% trichloroacetic acid (TCA).

The liver cytosolic fractions were obtained from livers of overnight fasted rats and used to measure the soluble antioxidant enzyme activities. Liver samples were homogenized in ice-cold medium containing 200 mM mannitol, 75 mM sucrose, 2.0 mM Tris, 0.2 mM EGTA, 100 *μ*M PMSF, and 50 mg% free bovine albumin fatty acids (pH 7.4) using a Dounce homogenizer (20 mL of the homogenization buffer per 2.5 g of tissue). These fractions were used to measure the activities of GPx3, GR, and copper/zinc-superoxide dismutase (Cu,ZnSOD). To measure the glucose 6-phosphate dehydrogenase (G6PD) activity, livers from fed animals were used and the samples were homogenized in medium containing 0.1 M Tris/HCl buffer and 1 mM EDTA, pH 7.6. All of these homogenates were centrifuged at 30,000 ×g for 15 min and the activities of these enzymes were determined in the supernatant using classical methods as briefly described below.

### 2.12. Protein Determination

Protein concentrations were determined according to the method of Lowry et al. [21] using bovine serum albumin as a standard.

### 2.13. Mitochondrial *β*-Oxidation Capacity

Liver mitochondrial *β*-oxidation was measured according to the amount of oxygen consumed by intact mitochondria during fatty acid oxidation through polarographic measurements using a Clark-type oxygen electrode (Yellow Springs Instruments, Yellow Springs, OH, USA) [22]. Mitochondria (0.6–1.2 mg/mL) were incubated in a closed chamber, where volumes of 2.0 mL of the incubation medium were maintained under agitation and warmed at 37°C through external water recirculation. The incubation medium contained 2.0 mM potassium phosphate monobasic, 0.1 mM EGTA, 130 mM potassium chloride, 5 mM magnesium chloride, 0.1 mM DNP, 2.5 mM L-malate, 10 mM HEPES (pH 7.2), and 50 mg% fatty acid-free bovine-serum albumin [22]. The reactions were initiated by the addition of (a) 20 mM octanoyl-CoA + 2.0 mM L-carnitine, (b) 20 mM palmitoyl-CoA + 2.0 mM L-carnitine, or (c) 20 mM palmitoyl-L-carnitine. The rate of oxygen consumption was expressed as nmol/min × mg of mitochondrial protein.

### 2.14. Peroxisomal *β*-Oxidation Capacity

The peroxisomal capacity to oxidize the long chain fatty acid, palmitate, was measured fluorimetrically using the method described by Small et al. [23] with minor modifications [24]. The assay was based on H_2_O_2_ production in a reaction catalyzed by exogenous peroxidase, which was coupled to the oxidation of DCFH-DA into the highly fluorescent compound DCF. The enzyme activity was monitored in real time by recording the variations in fluorescence. DCFH-DA was prepared daily in ethanol at a concentration of 5.0 mM and was kept on ice in the dark until use. Media (2 mL) containing 11 mM potassium phosphate monobasic, pH 7.4, 40 mM aminotriazole, 0.02% Triton X-100, and 150 *μ*g/mL horseradish peroxidase were added to a cuvette. The reaction was carried out at 30°C under constant agitation. A 25 *μ*M DCFH-DA solution was added after the addition of the peroxisome-enriched fraction (0.3 mg protein/mL). After three minutes, the reaction was initiated by the addition of palmitate, as an acyl-CoA derivative (final concentration of 30 *μ*M). The increase in fluorescence (excitation, 503 nm; emission, 529 nm) was recorded over a period of 10 min, and the activity of this fatty acyl-CoA oxidase was expressed as pmol DCF produced/min × mg peroxisomal protein. The concentrations of DCF produced in the reaction were calculated using a standard curve of DCF concentrations over a range of 50–1000 nM.

### 2.15. Mitochondrial ROS Generation

ROS production by fresh mitochondria was evaluated using DCFH-DA oxidation assays as previously described [25]. The real time formation of DCF was carried out at 30°C under agitation in a cuvette, where volumes of 2 mL of the incubation medium containing 250 mM mannitol, 40 mM aminotriazole, and 10 mM Hepes buffer (pH 7.2) were added. After a 5 min preincubation period of the mitochondrial suspensions (1 mg protein/mL), 5 mM succinate and 15 *μ*M DCFH-DA were added and this mixture was incubated for another 3 min. Thereafter, the rate of autooxidation drops considerably and the reaction was initiated by the addition of 600 *μ*mol/L ADP. Mitochondrial H_2_O_2_ generation was estimated by measuring the linear fluorescence increase (excitation, 503 nm; emission, 529 nm) recorded over a period of 10 min. Rates were then corrected for a substrate blank. Mitochondrial H_2_O_2_ generation was expressed as pmol DCF produced/min × mg mitochondrial protein.

### 2.16. Determination of GSH Contents in the Liver Homogenate and Isolated Mitochondria

GSH contents were measured fluorometrically using OPT according to the method described by Hissin and Hilf [26] with modifications. The supernatant of each fraction was added in a reaction medium containing 0.1 M phosphate buffer and 5.0 mM EDTA, pH 8.0. The reaction was started by adding 100 *μ*L of OPT solution (1 mg/mL, in methanol). OPT reacts with GSH at pH 8.0 to form a fluorescent product. The fluorescent product of this reaction, GSH-OPT, was measured fluorometrically (350 nm excitation and 420 nm emission) after an incubation period of 15 min at room temperature. The results were expressed as *μ*g GSH/mg protein present in the supernatant.

### 2.17. Determination of the Lipid Peroxidation Levels in the Liver Homogenates

TBARS were used as biomarkers of oxidative stress and lipid peroxidation [27] and were measured using direct spectrophotometry. The results were expressed as nmol of malondialdehyde (MDA)/mg protein using a molar extinction coefficient for MDA of 1.56 × 10^5^ M^−1^ cm^−1^.

### 2.18. Activities of Antioxidant Enzymes in Subcellular Fractions

CAT activity was assessed in the peroxisomal fractions, which concentrate the bulk of this enzyme activity [28, 29], and was expressed as H_2_O_2_ consumed/min × mg protein.

The activities of the two isoforms of GPx were assessed in the cytosolic (GPx3) fractions or in mitochondrial matrix (GPx1) according to their ability to oxidize GSH in the presence of H_2_O_2_. The reaction was performed in a cuvette at 22°C, and the decrease of absorbance at 340 nm was recorded over a period of 90 seconds [30]. The activity of this enzyme was expressed in nmol of NADPH oxidized/min × mg protein (*ε*, 6,220 M^−1^ × cm^−1^).

The activity of GR was determined according to the decrease in absorbance at 340 nm due to NADPH oxidation [31] in the cytosolic and mitochondrial fractions. The decrease in absorbance due to the consumption of NADPH was measured for 90 seconds. The activity of this enzyme was expressed in nmol of NADPH oxidized/min × mg protein (*ε*, 6,220 M^−1^ × cm^−1^).

The activity of SOD was measured according to its ability to inhibit the autoxidation of pyrogallol. In the liver, MnSOD is located in the mitochondrial matrix, whereas Cu,ZnSOD is more broadly distributed in almost all other compartments and is preferentially located in the cytosol as a soluble enzyme [19, 32]. These two isoforms can be distinguished from each other according to the sensitivity of Cu,ZnSOD to cyanide. The rate of increase in the absorbance at 420 nm was recorded for three minutes in a spectrophotometer, in the absence and presence of KCN 2.0 mM. One unit of SOD activity (U) was defined as the amount of the enzyme required to inhibit the rate of pyrogallol autoxidation by 50%; therefore, the enzymatic activity was expressed as U of SOD/mg protein [33].

The activity of G6PD was determined spectrophotometrically by measuring the rate of increase in absorbance [34] at 340 nm, due the conversion of NADP^+^ to NADPH by G6PD, using the molar extinction coefficient of NADPH.

The activity of the enzyme NNT was measured in isolated liver mitochondria by direct spectrometry using a combination of previously described methods with modifications [35, 36]. The reaction medium contained 50 mM Tris (pH 8.0), 0.5% Brij L23 solution, 1 mg/mL 1-Palmitoyl-*sn*-glycero-3-phosphocholine, 150 *μ*M APAD, and 150 *μ*M NADPH at a final volume of 1 mL. The reaction was initiated by the addition of mitochondrial suspensions (50 *μ*g/mL). APAD, an analog of NAD^+^, was used instead of NAD^+^ due the difference in the absorption spectrum of APADH compared with NADPH and NADH. This enables us to evaluate the rate of APADH formation in real time according to the increase of absorbance at 375 nm, which was recorded for 3 min. The rates of APAD reduction were expressed as nmol APADH produced/min × mg protein using the molar extinction coefficient of APAD of 5.1 mM^−1^ × cm^−1^.

### 2.19. Treatment of Data

The data in the figures and tables are presented as the means ± standard error of the mean (SEM). The data were analyzed using analysis of variance (ANOVA), followed by the Newman-Keuls posttest. The compared values are provided in the text as probability values (*P*), and the minimum criterion of significance was *P* ≤ 0.05. Statistical analyses were performed with Prism GraphPad 5.0 software (GraphPad Software, Inc.).

## 3. Results

### 3.1. General Features

As shown in Table 1, the pronounced uterine atrophy observed in the OVX groups indicated the success of the surgical procedure and the establishment of an estrogen-deficient condition in these animals. All OVX rats exhibited higher body weight gain than control animals, approximately 20%, which could not be attributed to differences in food ingestion. Despite the similar body weight gain, only the untreated OVX rats exhibited an adiposity index that was significantly higher than that of the control animals (+30%, approximately). In these animals, with the exception of uterine fat, all other fat deposits were increased; the mesenteric, inguinal, and retroperitoneal fats increased 24%, 44%, and 35%, respectively, over those of the control rats. Therefore, CE and ButF treatment suppressed the adiposity index of the OVX rats without a reduction in the body weight.

### 3.2. Serum and Plasma Biochemical Analysis

Table 2 shows that the lipid profile and glucose levels were not significantly modified by ovariectomy or the treatment of OVX rats with CE and ButF.

### 3.3. Liver Histochemical Analysis and Total Lipid Content

**Figure 2:**
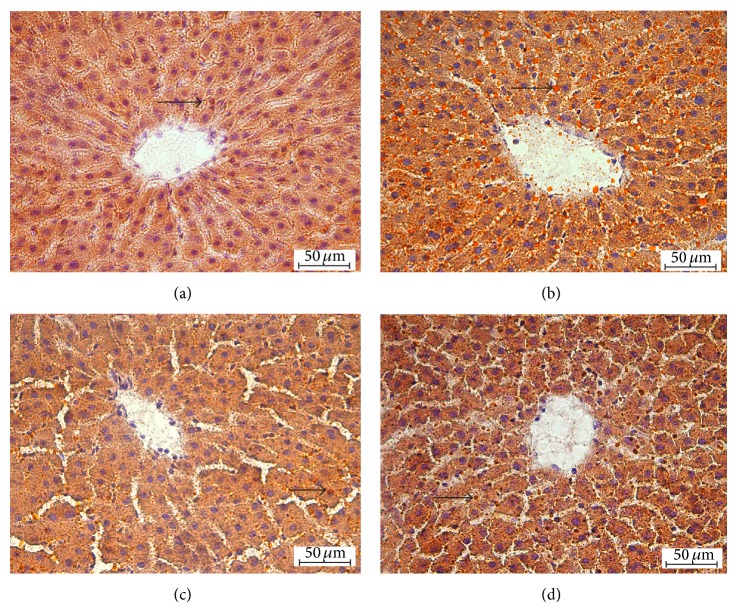
Liver histochemical analysis. Rat liver slices were frozen at −80°C, sectioned with a cryostat and stained for lipids using Sudan III. The images were captured at 40x magnification. In contrast to the control (panel (a)), OVX + CE (panel (c)), and OVX + ButF (panel (d)), the liver of OVX (panel (b)) contained considerable amounts of lipid inclusions (orange) as indicated by the arrows. Calibration bar: 50 *μ*m.

The liver histological sections of control (a), OVX (b), OVX + CE (c), and OVX + ButF (d) rats, stained with Sudan III, are presented in Figure 2. The untreated OVX rats displayed more lipid inclusions (in orange) than the control animals, and the livers from the OVX rats treated with CE and ButF have lipid inclusions similar to those of the controls.

**Figure 3:**
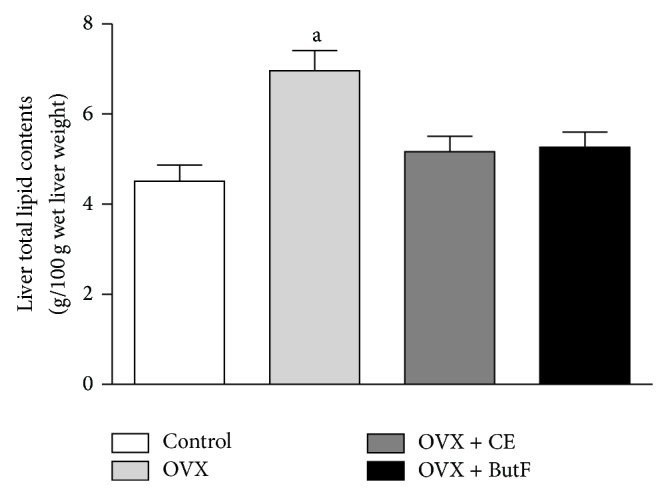
Gravimetric determination of liver total lipid content in liver. The liver fragments (approximately 1.0 g) from control, OVX, OVX + CE, and OVX + ButF rats were homogenized in a 2 : 1 chloroform-methanol mixture for the gravimetric determination of hepatic total lipids and the results are expressed as g/100 g wet liver weight (*n* = 6-7). The vertical bars represent the standard errors. The letter indicates significant differences between the values as calculated via ANOVA (^a^ *P* < 0.001 OVX* versus* control, OVX + CE, and OVX + ButF).

In agreement with these results, the quantification of the liver total lipid contents, performed by the gravimetric method (Figure 3), revealed that the livers from control rats presented normal total lipid levels (4.510 ± 0.36 g/100 g). The livers from untreated OVX rats, on the other hand, exhibited amounts significantly higher of total lipids, of approximately 35%, a characteristic of extensive steatosis, which was totally reversed by treating the animals with CE and ButF.

### 3.4. Liver Mitochondrial and Peroxisomal *β*-Oxidation Capacities

**Figure 4:**
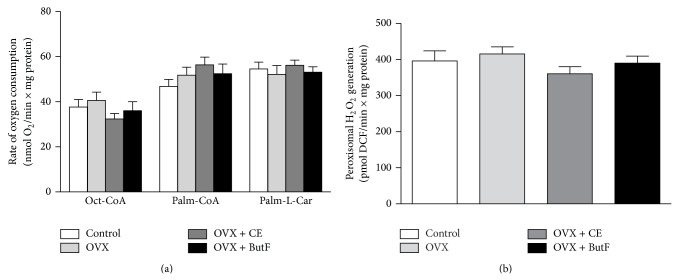
Mitochondrial and peroxisomal capacity to oxidize fatty acids. The liver mitochondrial *β*-oxidation capacity (panel (a)) was determined polarographically in the presence of 100 *μ*M 2,4-DNP. Mitochondria (0.6–1.0 mg/mL) were incubated in a total volume of 2.0 mL. Reactions were initiated by the addition of the following: 20 *μ*M octanoyl-CoA + 2.0 mM L-carnitine (Oct-CoA), 20 *μ*M palmitoyl-CoA + 2.0 mM L-carnitine (Palm-CoA), or 20 *μ*M palmitoyl-L-carnitine (Palm-L-Carn). The peroxisomal palmitoyl-CoA oxidase activity (panel (b)) from control, OVX, OVX + CE, and OVX + ButF rats were measured via fluorimetry (excitation, 503 nm; emission, 529 nm) based on the oxidation of DCFH-DA by H_2_O_2_ into DCF in a reaction catalyzed by exogenous peroxidase. The reactions were initiated by the addition of 30 *μ*M palmitoyl-CoA (palm-CoA). The values are expressed as the means of 6–10 individual experiments with different mitochondrial and peroxisomal preparations. The vertical bars represent the standard errors and statistical significance was evaluated using ANOVA (*P* < 0.05).

Figure 4 (panel (a)) shows the ability of liver mitochondria isolated from control, OVX + CE, and OVX + ButF rats to oxidize octanoyl- and palmitoyl-CoA (in the presence of L-carnitine) and palmitoyl-L-carnitine. In panel (b), the liver peroxisomal *β*-oxidation is shown. As shown, fatty acid oxidation in both organelles was not different between the four groups of animals.

### 3.5. The Liver Mitochondrial Redox State

**Figure 5:**
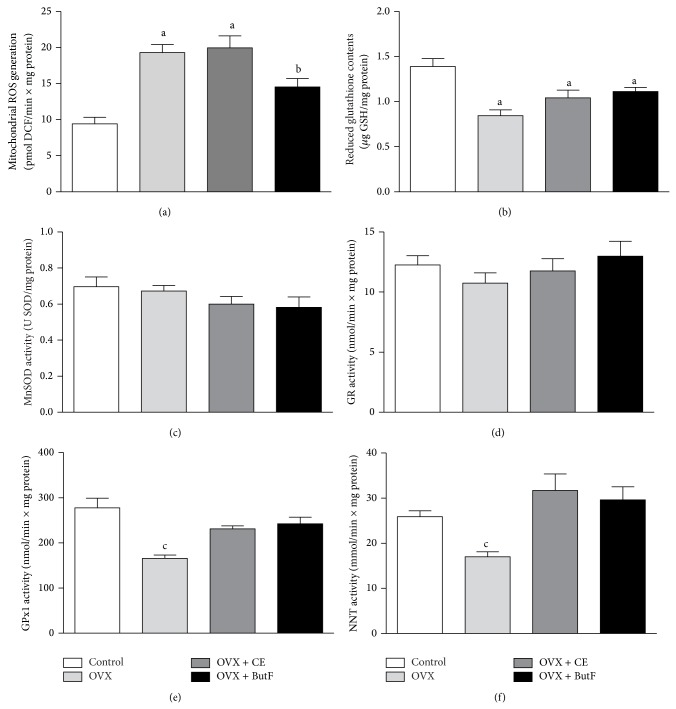
Mitochondrial ROS generation and ROS scavenger systems. The liver mitochondrial oxidative status was evaluated by assessing mitochondrial ROS generation and the ROS scavenging system. Mitochondrial H_2_O_2_ generation (panel (a), pmol DCF/min × mg protein; *n* = 5–8); mitochondrial GSH levels (panel (b), *μ*g GSH/mg protein; *n* = 6–10); MnSOD activity (panel (c), U SOD/mg protein; *n* = 5-6); mitochondrial GR activity (panel (d), nmol NADPH oxidized/min × mg protein; *n* = 7–9); GPx1 activity (panel (e), nmol NADPH oxidized/min × mg protein; *n* = 6–8), and NNT activity (panel (f), mmol APADH produced/min × mg protein; *n* = 4-5) were evaluated. The vertical bars represent the standard error. The letters indicate significant differences between the values as determined by ANOVA (*P* < 0.05): letter a indicates the mean values different from the control; letter b indicates the mean values different from the OVX and OVX + CE; letter c indicates the mean values different from the control, OVX + CE, and OVX + ButF.

Mitochondria are the main cellular source of ROS production, and alterations in the liver redox status could be a result of alterations in mitochondrial ROS generation, a possibility that was evaluated in this work. As observed in Figure 5 panel (a), H_2_O_2_ production in liver mitochondria from the OVX rats was significantly higher than that of the control animals (+51%). The CE treatment of the OVX rats did not modify the mitochondrial production of H_2_O_2_ in comparison with the mitochondria from the untreated-OVX rats, but a significant reduction (−64%) was found in the mitochondria isolated from the ButF-treated animals (OVX + ButF).

Mitochondria possess several mechanisms to scavenge ROS, including antioxidant compounds, such as GSH, and many enzymes. Figure 5 panel (b) shows that the mitochondrial content of reduced glutathione (GSH), which was significantly reduced in the OVX rats (−39%), was not restored by either treatment in the OVX animals.

The activities of antioxidant enzymes were also assessed in isolated mitochondria: MnSOD (panel (c)), GR (panel (d)), GPx1 (panel (e)), and NNT (panel (f)). However, only GPx1 activity and NNT activity were modified in the experimental groups. The activity of both enzymes, which were reduced in liver mitochondria from the OVX animals, increased to values close to those of the control rats in mitochondria isolated from the OVX rats treated with both CE and ButF.

In these experiments with mitochondria, the MnSOD activities were assessed in two different experimental conditions: in the absence and presence of 2.0 mM potassium cyanide (KCN). The results presented here were performed in the presence of KCN, which, however, did not differ significantly from those performed in the absence of this inhibitor (data not shown), demonstrating that the contamination with Cu,ZnSOD was minimal.

### 3.6. Liver Redox Status

**Figure 6:**
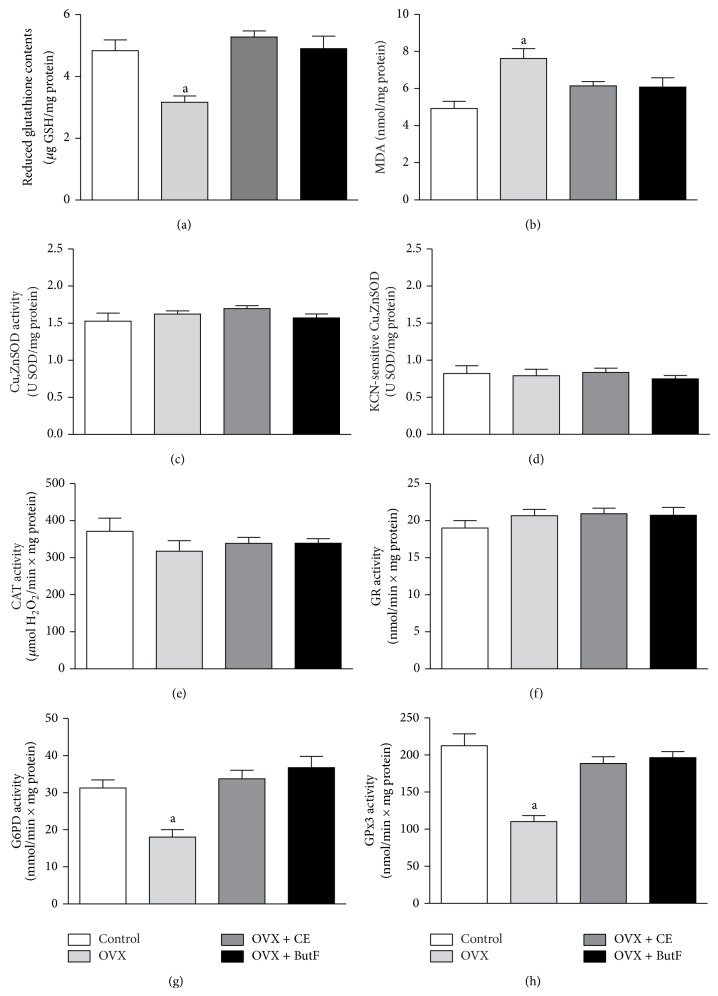
Evaluation of the liver redox status. The liver oxidative status was evaluated by assessing the liver GSH levels. GSH (panel (a), *μ*g GSH/mg protein; *n* = 6–10); liver lipid peroxidation using the TBARS method (panel (b), nmol MDA/mg protein; *n* = 7–9); total Cu,ZnSOD activity (panel (c)) and KCN-sensitive Cu,ZnSOD (panel (d)) were measured and expressed as U SOD/mg protein (*n* = 5-6); CAT activity (panel (e), *μ*mol H_2_O_2_ consumed/min × mg protein; *n* = 6–10); GR activity (panel (f), NADPH oxidized/min × mg protein; *n* = 7–9); G6PD activity (panel (g), NADPH produced/min × mg protein; *n* = 6–9) and GPx3 activity (panel (h), NADPH oxidized/min × mg protein; *n* = 7–9) were evaluated. The vertical bars represent the standard error. The letters indicate significant differences between the values as determined using ANOVA (^a^ *P* < 0.05 OVX* versus *control, OVX + CE, and OVX + ButF).

The increased mitochondrial H_2_O_2_ production found in the OVX rats can disturb the entire cellular redox status. GSH and antioxidant enzymes are also present in other cell compartments. Some of these enzymes and biomarkers of the liver redox status were then measured in liver fractions, and the results are presented in Figure 6 (panels (a) to (h)). GSH levels (panel (a)), which were significantly reduced in the OVX rats, were completely restored with CE and ButF treatment. In agreement with this, the lipid peroxidation levels (panel (b)), which were increased in the OVX animals, returned to values similar to those found in the control animals with CE and ButF treatment.

The evaluation of the most important enzymatic scavenger system revealed that neither total Cu,ZnSOD (panel (c)) nor the KCN-sensitive (panel (d)) activities were different in the four groups. Additionally, CAT activity (panel (e)) was not different in these groups.

The evaluation of the enzymes involved in the GSH cycle revealed that although the GR activity (panel (f)) was not different between the groups, the G6PD enzyme (panel (g)), which provides the reducing equivalents to GR, was reduced in the OVX rats and was completely recovered by CE and ButF treatment.

A similar pattern was exhibited by the GPx3 (panel (h)) enzyme, which was reduced in the OVX animals and returned to values similar to those of the control animals with CE and ButF treatment.

## 4. Discussion

In this study, the OVX rats were treated with a crude VAC extract or an* n*-butanolic fraction (ButF) and both exerted similar beneficial effects on the lipid metabolism and redox status of the liver.

The OVX rats exhibited increased body weight gain, increased abdominal fat deposits, and hepatic steatosis, as previously described by our group [3, 4] and other authors [1, 2, 5]. Our results also demonstrated that the increase in body weight gain was not due to an increase in food intake and that the rats were still normoglycemic and had no dyslipidemia 13 weeks after ovariectomy, which was also in accord with previous reports [3, 4].

CE and ButF treatment of the OVX rats reduced the adiposity index and the lipid accumulation in the liver, despite the observation that the rats retained a higher body weight. The fatty acid accumulation observed in the liver of the OVX rats could not be attributed to an overload of FFA derived from lipolysis in adipose tissues because the plasmatic level of FFA was not increased in the OVX rats. An imbalance between the hepatic capacities for synthesizing and oxidizing fatty acids is suggested to be an important causative factor of NAFLD [5]. However, our present work indicated that neither the development of steatosis in the OVX rats nor its reversion by CE and ButF treatment could be attributed to a reduction in the liver's capacity to oxidize FFA via the mitochondrial or peroxisomal pathways. These results are in agreement with our previous work [3, 4]. Future studies should investigate the role of altered hepatic TG synthesis [37] and/or export in liver fat accumulation. Indeed, there is report showing reduced export of TG as VLDL-cholesterol from the liver of estrogen-deficient animals [38].

Most of the alterations observed in the untreated-OVX rats may be consequences of estrogen deficiency, which often leads to metabolic dysregulation [1]. Mitochondria also respond to estrogens through alpha and beta receptors [39], and a previous study showed that liver mitochondria isolated from estradiol-treated male rats produced less ROS [40]. Estrogen can also upregulate the nuclear expression [41] and/or the activity [3, 4] of several antioxidant enzymes.

The findings that CE and ButF treatment of the OVX rats reduced the adiposity index and steatosis are very important beneficial properties of these extracts because it is well known that an overload of FFA in the hepatocyte cytosol induces oxidative cellular damage [6]. Moreover, in the liver, oxidative stress plays a central role in the progression of NAFLD to more severe forms of the disease, such as fibrosis and cirrhosis [42, 43].

We have actually found that the steatosis in the livers of the untreated OVX was associated with higher production of mitochondrial ROS and the characteristic signs of oxidative stress, including lower mitochondrial and cellular GSH, increased MDA levels, and reduced activity of the antioxidant enzymes GPx, G6PD, and mitochondrial GR, also confirming our previous work [3, 4].

Despite the complete reversion of the steatosis by CE and ButF treatment of the OVX rats, only the agnuside-enriched fraction was partially effective in reducing mitochondrial ROS generation.

The cellular consequences of higher mitochondrial ROS production, however, will depend on the antioxidant systems operating in other cellular compartments [44–47]. In mitochondria, the redox cycle of GSH is a major endogenous antioxidant system that provides protection against ROS, which is why these organelles are so sensitive to reductions in their GSH pools [48]. Although the mitochondrial ROS generation was partially reduced by CE and ButF treatment, the GSH levels were not reestablished with treatment. These results suggested that the mitochondrial capacity to dispose of H_2_O_2_, which depends on a balance between the activity of MnSOD and the GSH redox cycle, has been impaired [49]. Because the activity of MnSOD in the untreated or treated OVX rats was unaffected, this raised the possibility that the enzymes involved in the GSH redox cycle were altered.

The evaluation of the activities of GR, the GSH-restorer enzyme, and NNT, which provides NADPH used by mitochondrial GR [35], revealed that the activity of GR was not affected but that the low levels of NNT activity in the OVX rats were restored in the animals treated with each extract. Therefore, the lack of a restoration in the GSH levels in the treated animals could not be explained on this basis, despite the partial reduction in H_2_O_2_ generation. However, the evaluation of GPx1 activity, the major mitochondrial GSH-consumer enzyme, produced interesting results. GPx1 activity was reduced in the OVX rats and was restored in the treated animals. This, associated with the increased ROS generation still observed in these animals, could explain, at least in part, the diversion of the GSH redox cycle toward the direction of the oxidized state.

Different from mitochondria, however, the cytosolic GSH contents that were reduced in the OVX animals were completely restored by treating these animals with both extracts. This effect could not be explained on the basis of alterations in the activity of Cu, ZnSOD, or CAT, which remained unaffected in all of the animals. However, the GPx3 activity, which was reduced in the OVX rats, was restored in the treated animals, similar to mitochondrial GPx1.

As in mitochondria, the GR activities [50] were also similar in all animal groups, as expected. However, the G6PD was reduced in the OVX rats and was completely restored in the treated animals, which could be a contributing factor to GSH restoration in these animals. The fact that the G6PD expression and activity are positively regulated by estrogen has been well-described [51] and was recently demonstrated in our previous study [3, 4].

With respect to liver GPx3 activity, there are reports that it is reduced in estrogen-deficient conditions [52]. However, this effect appears to be time-dependent [53]. Recent studies performed in our lab [3, 4] revealed that the activity of this enzyme was unaffected after a period of 5 weeks, although a clear tendency toward reduced activity has been observed in OVX animals.

The sum of the changes found in the antioxidant enzyme activities measured in different cellular compartments revealed that despite not being able to suppress the higher production of mitochondrial ROS generation and to restore the mitochondrial GSH contents, the treatment of OVX rats with both extracts was able to avoid the ROS-induced damage to membranes, as indicated by reduction in the MDA content of the liver. This was most likely due to restoration of the activity of enzymes affected by ovariectomy that are located in all cellular compartments, which also promoted the normalization of GHS levels in the liver cells.

Although the estrogen deficiency could be related to most of the changes observed in the untreated-OVX rats, it is unlikely that the beneficial effects of the active components of VAC, for example, the AGN, were due to an estrogenic activity [54]. As a glycoside, AGN cannot bind to estrogenic receptors. The ability to protect the liver against oxidative stress was likely due to the antioxidant properties, not only of AGN but also of many other substances present in the extracts, including flavonoids and polyphenols [11, 55–57] that have recognized antioxidant properties.

It was demonstrated by Kadir et al. [58] that the ethanolic extract of* Vitex negundo,* which also contains AGN and many other phenolic components, exhibits strongest free radical scavenging power when compared to the standard antioxidants butylated hydroxytoluene and ascorbic acid. The antioxidant property of phenolic compounds is suggested to be related to the hydroxyl groups attached to aromatic rings [58]. Using the Prediction of Activity Spectra for Substances (PASS) software [59], Kadir et al. [58] demonstrated that AGN has predictable biological activity of lipid peroxidase inhibitor, antioxidant, free radical scavenger, hepatoprotectant, caspase-3 stimulant, and antiproliferative. This property could explain the capacity of ButF, which is enriched in AGN in reducing the mitochondrial H_2_O_2_ generation.

It should be noted that the ButF administered to the rats had 1.6-fold more AGN than the CE. The higher concentration of AGN in the ButF could explain the inability of CE to suppress ROS production in isolated mitochondria. However, the observed responses to CE or ButF treatment for all other measured parameters were very similar. There is no information regarding the intestinal assimilation of the active compounds of the extracts and their circulating concentrations. It is possible that AGN and other active compounds present in the extracts reached similar concentrations inside the hepatocytes, and, therefore, exerted similar effects. Further studies will be needed to clarify this hypothesis.

It can be concluded that the CE or ButF treatment of OVX rats was effective in preventing NAFLD and oxidative stress, which are frequent causes of abnormal liver functions in the postmenopausal period [1, 12, 13, 60].

## Figures and Tables

**Table 1:** General features.

	Control	OVX	OVX + CE	OVX + ButF
Uterine weight	0.117 ± 0.006^a^	0.014 ± 0.001	0.015 ± 0.001	0.015 ± 0.001
Body weight gain	116.8 ± 6.814^b^	150.4 ± 4.112	151.1 ± 5.206	142.3 ± 5.822
Food consumption	19.52 ± 0.266	20.87 ± 0.431	19.82 ± 0.514	19.58 ± 0.948
Adiposity index	6.205 ± 0.437	9.005 ± 0.661^c^	6.502 ± 0.211	7.040 ± 0.445
Mesenteric fat	1.096 ± 0.086	1.450 ± 0.092^d^	0.978 ± 0.073	0.892 ± 0.051
Uterine fat	0.558 ± 0.055	0.611 ± 0.021	0.598 ± 0.013	0.588 ± 0.074
Inguinal fat	1.778 ± 0.116	3.153 ± 0.268^e^	2.121 ± 0.198	2.532 ± 0.175^f^
Retroperitoneal fat	2.712 ± 0.355	4.150 ± 0.261^g^	2.991 ± 0.258	3.212 ± 0.303

Notes: the uterine and fat depots weights were expressed in g/100 g BW (*n* = 4–6). Thebody weight gain was expressed in g (*n* = 6–9). Food consumption was expressed in g/day (*n* = 6–9). The adiposity index was calculated from the sum of the retroperitoneal, uterine, mesenteric, and inguinal fat weights, which was related to g/100 g BW (*n* = 5). The results were expressed as the means ± SEM. The letters indicate the statistical significance as revealed by ANOVA (^a^ *P* < 0.005 control versus OVX, OVX + CE, and OVX + ButF; ^b^P < 0.05 control versus OVX, OVX + CE, and OVX + ButF; ^c^ *P* < 0.0001 OVX versus control, OVX + CE, and OVX + ButF; ^d^ *P* = 0.001 OVX versus control, OVX + CE, and OVX + ButF; ^e^ *P* = 0.001 OVX versus control, OVX + CE, and OVX + ButF; ^f^ *P* = 0.001 OVX + ButF versus control; ^g^ *P* < 0.05 OVX versus control and OVX + CE).

**Table 2:** Serum and plasma biochemical analysis.

	Control	OVX	OVX + CE	OVX + ButF
Triacylglycerols	29.95 ± 2.359	33.80 ± 2.600	30.61 ± 1.913	29.33 ± 1.950
Total cholesterol	70.79 ± 4.689	76.08 ± 2.663	76.25 ± 1.986	74.34 ± 3.776
HDL-cholesterol	35.97 ± 2.263	35.67 ± 1.647	36.27 ± 1.592	35.29 ± 1.617
LDL-cholesterol	24.56 ± 1.431	34.00 ± 2.932	33.04 ± 1.463	32.01 ± 3.104
VLDL-cholesterol	6.336 ± 0.527	6.411 ± 0.570	6.121 ± 0.382	5.601 ± 0.498
FFA	0.971 ± 0.072	0.836 ± 0.076	0.924 ± 0.046	0.845 ± 0.044
Glycemia	106.7 ± 4.063	110.5 ± 2.819	113.3 ± 4.213	109.6 ± 2.812

Notes: triacylglycerols (mg/dL; n = 6–9), total cholesterol (mg/dL; n = 6–10), high-density lipoprotein (HDL-cholesterol; mg/dL; n = 6–10), low-density lipoprotein (LDL-cholesterol; mg/dL; n = 5–10), very low density lipoprotein (VLDL-cholesterol; mg/dL; n = 7–11), free fatty acids (FFA; mg/dL; n = 8), and glycemia (mg/dL; n = 8–10) were expressed as the means ± SEM.
